# *Parageobacillus* and *Geobacillus* spp.: From Food Spoilage to Beneficial Food Applications

**DOI:** 10.3390/foods14162775

**Published:** 2025-08-09

**Authors:** Maika Salvador, Santiago Condón, Elisa Gayán

**Affiliations:** Department of Animal Production and Food Science, AgriFood Institute of Aragon (IA2), Faculty of Veterinary, University of Zaragoza-CITA, Miguel Servet 177, 50013 Zaragoza, Spain; msalvador@unizar.es (M.S.); scondon@unizar.es (S.C.)

**Keywords:** thermophilic microorganisms, bacterial spores, antibiotic detection test, valorization of agri-food residues, thermoenzymes

## Abstract

The genera *Parageobacillus* and *Geobacillus* comprise thermophilic, spore-forming bacteria. The extraordinary heat resistance of their spores, together with their ability to form biofilms and produce thermostable enzymes, makes them a relevant cause of spoilage in shelf-stable, heat-treated products like dairy and canned foods. However, these same biological traits offer valuable opportunities for the food industry. In this context, the purpose of this review is to describe the challenges posed by (*Para*)*Geobacillus* spp. as food spoilage agents, while also highlighting their existing and prospective applications in the food industry. In terms of food safety, *G. stearothermophilus* spores are used as biological indicators in commercially available tests to detect antibiotic residues in food within a few hours. Additionally, (*Para*)*Geobacillus* can be exploited for the fermentation of agri-food residues to produce high-value compounds such as biofuels, food ingredients and technological adjuvants, and compost. Their thermostable enzymes—such as amylases, xylanases, L-arabinose isomerases, β-galactosidases, lipases, proteases, and L-asparaginases—have potential applications in food processing and ingredient production. However, several challenges persist, including limited knowledge on genetic diversity, physiology, and metabolism, as well as low yields of biomass and target compounds. These issues reinforce the need for further studies to unlock their full potential.

## 1. Introduction

The genera *Parageobacillus* and *Geobacillus* (hereafter collectively referred to as (*Para*)*Geobacillus*) comprise Gram-positive, aerobic or facultative anaerobic, catalase-positive, spore-forming bacteria, well-known for their thermophilic traits. Depending on the strain, they can grow across a broad temperature range from 35 °C to 80 °C [1], although most require temperatures between 45 °C and 70 °C [2]. The genera include several relevant species, such as *G. stearothermophilus*, *G. thermodenitrificans*, *G. kaustophilus*, *P. caldoxylosilyticus*, *P. toebii*, and *P. thermoglucosidasius*, among others. Both genera were originally part of the *Geobacillus* genus, which was formerly separated from the thermophilic *Bacillus* group based on 16S rRNA gene sequence analysis [2]. Subsequent phylogenetic advances based on core genome analysis led to the reclassification of the *Geobacillus* genus into the following two clades differentiated by nucleotide composition: clade I, corresponding to *Geobacillus* genus, with a G+C content of 48.8–53.1%; and clade II, corresponding to *Parageobacillus* genus, with a lower G+C content of 42.1–44.4% [3,4,5].

**Figure 1:**
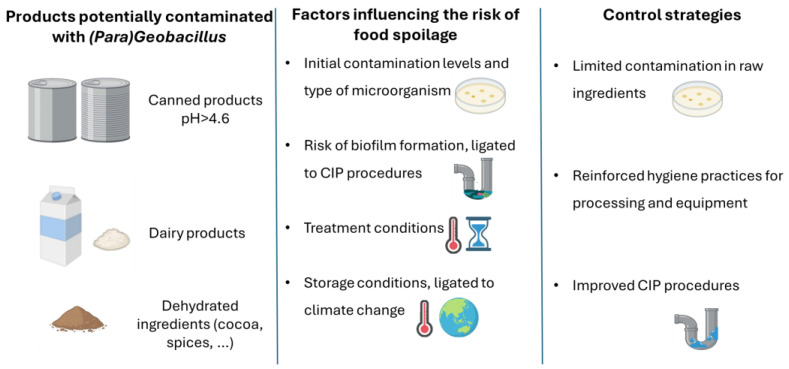
Schematic overview of the presence of (*Para*)*Geobacillus* spp. in food systems, along with the main factors influencing their persistence and the corresponding control measures. CIP refers to cleaning-in-place procedures. Created in BioRender. Salvador, M. (2025) (www.biorender.com) and Freepik (www.freepik.com).

Both genera typically inhabit hot environments such as equatorial deserts, hydrothermal vents, and hot springs, but they can be widely found in nature, even in cold soils and ocean sediments, likely due to their spore-forming capability [6]. Owing to their ubiquity, spores can easily contaminate food materials and once introduced into processing facilities they can colonize hot surfaces and form biofilms, particularly in bulk-treated products, becoming a persistent source of spores and spoilage enzymes [7,8,9]. Spores from some (*Para*)*Geobacillus* spp., such as *G. stearothermophilus*, are extraordinarily heat-resistant and can survive intense thermal sterilization or Ultra-High Temperature (UHT) treatments [10]. Combined with contamination by their thermostable spoilage enzymes, this poses a threat to the quality and economic viability of shelf-stable, heat-treated foods—especially low acid canned foods (pH > 4.6) and dairy products [11,12,13]. Figure 1 illustrates the integration of these species into the food industry, highlighting the key factors influencing their survival and the control strategies that are further discussed throughout this review.

**Figure 2:**
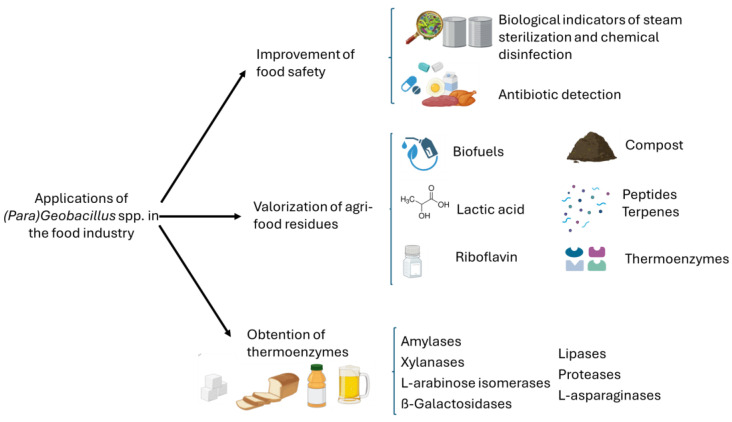
Schematic overview of the potential applications of (*Para*)*Geobacillus* spp. in the food industry, including improvement of food safety, valorization of agri-food residues, and production of thermostable enzymes. Created with BioRender. Salvador, M. (2025) (www.biorender.com) and Freepik (www.freepik.com).

On the other hand, (*Para*)*Geobacillus* spp. offer notable advantages for beneficial applications in the food industry (Figure 2). Figure 2 highlights the diverse applications of (*Para*)*Geobacillus* spp., including the improvement of food safety, valorization of agri-food residues, and the production of thermostable enzymes with potential industrial uses. Food safety remains a critical concern, and spores from these genera have been used as commercial biological indicators to verify the effectiveness of sterilization processes by heat and chemicals, such as hydrogen peroxide [14,15]. In addition, the high stability of their spores combined with rapid germination and growth at optimal temperatures and sensitivity to a broad spectrum of antimicrobials makes them suitable biological sensors for detecting antibiotic residues in food. Furthermore, their ability to thrive at elevated temperatures and their capacity to produce a variety of enzymes that degrade agri-food residues into fermentable feedstocks make them attractive candidates for use as cell factories in the production of various high-value compounds, including biofuels and food ingredients such as lactic acid, riboflavin, terpenes, and oligosaccharides [16,17,18]. Finally, they are also an important source of thermostable enzymes with a variety of applications in the food industry, such as amylases for baking, sweetener production, or brewing [19,20,21,22,23,24,25,26,27,28,29,30], and proteases for the synthesis of aspartame precursors or functional bioactive peptides [31,32].

This review aims to examine the challenges associated with (*Para*)*Geobacillus* spp. as food spoilage microorganisms (Figure 1), while also exploring their current and potential applications in the food industry based on their advantageous biological traits (Figure 2).

## 2. Food Spoilage

(*Para*)*Geobacillus* spp. spores can be found in a wide variety of products that undergo shelf-stabilizing thermal treatment and/or dehydration processes, where quality guidelines encompass the enumeration of thermophilic spores as an indicator of food stability or process hygiene [33,34]. These include vegetable products (peas, mixed vegetables, green beans, soups, spinach, etc.), dairy products (UHT milk, milk, and whey powders), plant-based dairy alternatives, sauces, ready meals (poultry, cassoulet, cottage pie, red meat, ravioli, quenelles, etc.), and ingredients (cocoa powder, spices, gelatin, etc.) [11,12,35,36,37,38,39].

Despite their widespread distribution, (*Para*)*Geobacillus* spp. spores pose a spoilage risk only in foods with permissive conditions of germination and outgrowth, including pH values around 6.0–8.0, water activity (a_w_) between 0.985 and ≥0.999, and salt concentrations below 4.0% [13,40,41]. Spoilage typically occurs when products are stored at temperatures above 35 °C, the minimum growth temperature reported for *G. stearothermophilus* [42]. Due to climate change, the risk of spoilage in shelf-stable products caused by thermophilic spores is expected to increase in the coming years, particularly in Southern European countries during the summer months [38,42,43,44].

*G. stearothermophilus* is the primary species responsible for spoilage in products processed in-pack. In a ten-year survey conducted in France, (*Para*)*Geobacillus* spp. were responsible for 35% of canned food spoilage cases. Among these, *G. stearothermophilus*, widely known for causing flat sour spoilage, accounted for 94% of them, while the remaining cases involved other species such as *P. caldoxylosilyticus* and *P. thermoglucosidasius* [12].

In addition, *G. stearothermophilus* is particularly challenging in bulk-processed dairy products, especially in milk and milk-derived powders. In these foods, contamination with thermophilic spores dramatically increases in the end product, up to reach levels as high as 10^6^ CFU/g [45,46], compared to raw milk (<10 CFU/mL) [47,48]. *G. stearothermophilus*, *Anoxybacillus flavithermus*, and *Bacillus licheniformis* are the most predominant thermophilic spores [46,47,49,50], with the former constituting up to 50% of the identified isolates in some plants [51,52]. While the presence of *B. licheniformis* spores—a mesophilic bacilli capable of growing at temperatures as high as 60 °C [48]—has been associated with raw milk contamination [47], spores of the obligate thermophilic (*Para*)Geobacilli and Anoxybacilli genera are likely to originate from biofilms on manufacturing surfaces [53]. Biofilm formation poses a major challenge for the dairy industry, becoming a persistent source of contamination not only with highly resistant spores but also with spoilage enzymes in the final product [7,8,9]. Over the past decades, several research efforts have been dedicated to understanding the biofilm formation of thermophilic spore-formers in dairy processing systems and to developing corrective measures [53,54], as described below.

Spoilage of cocoa-containing milk by thermophilic spores has been reported, although the main source of contamination remains unclear [55,56,57,58,59]. The occurrence of thermophilic spores such as (*Para*)*Geobacillus* spp. in cocoa powder is occasional and usually at extremely low concentrations (<10 CFU/g) [55,56]. However, some authors have reported higher contamination levels of thermophilic aerobic spores in unroasted cocoa beans or nibs, reaching around 3.0 log CFU/g, which could survive thermal processing and cause spoilage [60]. Moreover, during cocoa fermentation, temperatures can reach up to 50 °C, creating favorable conditions for the growth and sporulation of *G. stearothermophilus,* thereby increasing the risk of spoilage in products made from cocoa powders [55,61].

### 2.1. Biofilm Formation by (Para)Geobacillus spp. as a Source of Microbial and Enzymatic Contamination in the Dairy Industry

As mentioned, (*Para*)*Geobacillus* spp. and *A. flavithermus* predominate in biofilms in milk processing facilities operating at elevated temperatures (40–65 °C), such as evaporators and plate heat exchangers [50,62]. Despite their typically low levels in raw milk, vegetative cells and spores can adhere to milk-contact surfaces, where they rapidly multiply. Under optimal conditions, these microorganisms have a doubling time between 15 and 35 min [51,63,64], and can form a fixed-state biofilm within 6–24 h [63,64,65].

Several factors influence cell attachment and biofilm formation by (*Para*)*Geobacillus* spp., including surface material and topography, temperature, flow rate, oxygen availability, milk composition, and cleaning procedures [54,62,66,67]. Thermophilic bacteria preferentially form biofilms on stainless steel or glass rather than food-grade plastics, and they are more likely to colonize air-liquid interfaces, such as partially filled piping systems exposed to oxygen, than fully submerged surfaces [50,62,64]. Interestingly, *Anoxybacillus* spp. are a major concern for biofilm formation in the presence of skim milk, whereas (*Para*)*Geobacillus* spp. are more commonly associated with whole milk products [62]. Biofilm formation by *Geobacillus* spp. may be influenced by variations in ion concentrations, including sodium, calcium, and magnesium [65]. The deposition of organic material and salts on surfaces, known as fouling, plays a critical role in promoting biofilm development by thermophilic bacteria [54,66]. Vegetative cells and spores of *G. stearothermophilus* adhere to surfaces coated with denatured whey proteins at levels more than 100 times higher than those observed on clean surfaces [63]. The simultaneous occurrence of fouling and biofilm formation, referred to as biofouling, can shield spores from cleaning and disinfection agents [68,69]. This highlights the importance of implementing efficient and frequent cleaning-in-place (CIP) protocols [69]. However, it is worth noting that exposure of *G. stearothermophilus* spores to NaOH alters their surface hydrophobicity and negative charge, thereby improving their adhesion to stainless steel [70].

Intraspecific variations have been reported in the surface properties of spores and their capacity to form biofilms [50,62,64,71]. In addition, the impact of the aforementioned external factors may vary among strains. For instance, the preferred surface material and temperature for achieving maximum cell densities in biofilms differ among *G. thermodenitrificans* strains [62], and the effect of calcium concentration on biofilm formation varies among *G. stearothermophilus* strains [72]. Furthermore, biofilms are often multi-species communities in which social interactions influence the species abundance and temporal dynamics, as well as their architecture and resistance properties [73]. The growth of *P. thermoglucosidasius* dairy isolates lacking lactose utilization genes in skim milk depends on the presence of *A. flavithermus* strains to supply intermediate metabolites [64,74]. In contrast, most *G. stearothermophilus* isolates are generally adapted to utilize lactose independently, enabling them to grow and form biofilms in milk without assistance [13,64]. Moreover, *G. stearothermophilus* has shown an antagonistic relationship with *A. flavithermus* in terms of biomass production within biofilms [75].

Spores released from biofilms can survive drying and thermal processing, and germinate in the final product at favorable temperatures, as in the case of powder products upon rehydration, leading to off-flavors and acidic coagulation [42,46,76]. Besides cellular contamination, planktonic or biofilm-associated growth of (*Para*)*Geobacillus* cells in processing facilities has been linked to the production of thermally stable enzymes. Notably, some *G. stearothermophilus* and *G. thermoleovorans* dairy isolates have shown lipase, protease, and β-galactosidase activities [7,8,9]. Lipases are responsible for rancidity and fruity flavors, while proteases produce bitter peptides that lead to rotten and bitter flavors in milk [7,8]. Proteases and, in particular, lipases produced by *G. stearothermophilus* have been shown to retain partial activity after heat treatments commonly applied during milk powder production [8]. These enzymes may be active at lower temperatures than those required for microbial growth [77,78,79]. Although it remains unclear whether these enzymes can deteriorate low-water-activity products during storage [53], they are likely to regain activity in reconstituted foods [8].

### 2.2. Strategies to Control (Para)Geobacillus spp. in Food

Controlling the microbiological quality of raw materials is the first crucial measure in minimizing the introduction of (*Para*)*Geobacillus* spp. into food production. Low-acid, high-water-activity products (pH > 4.6, a_w_ > 0.85) require minimal thermal processing to achieve a 12-log reduction in *Clostridium botulinum* spores to ensure food safety. However, this treatment does not always sufficiently inactivate heat-resistant spoilage microorganisms, including (*Para*)*Geobacillus* spp. spores [35]. *G. stearothermophilus* and *G. thermoleovorans* spores present extraordinary heat resistance to UHT treatments [7,80], with some *G. stearothermophilus* strains showing a 4.6-log reduction in milk after treatment at 125 °C for 30 min [80]. Moreover, extrinsic factors such as sporulation conditions [41] or insufficient hydration of powdered ingredients may favor spore survival [56].

It should be kept in mind that shelf-stabilizing thermal processes are typically optimized to target heat resistant mesophilic spores, which are more likely to germinate during storage at moderate temperatures, while surviving thermophilic spores may remain inactive [35,53]. However, as climate change threatens to increase the risk of spoilage by obligate thermophiles [38,42,43,44], and as intensifying thermal treatments may not be economically viable and/or may result in unacceptable quality loss [81], there is growing interest in the development of alternative strategies for inactivating thermophilic spores. Emerging processing technologies at elevated temperatures (85–105 °C), such as high hydrostatic pressure, ultra-high-pressure homogenization, or supercritical CO_2_, have been reported to effectively inactivate *G. stearothermophilus* spores at lower temperatures than those required by conventional thermal treatments [82,83,84]. Further research into the mechanisms of heat resistance in (*Para*)*Geobacillus* spp. spores may facilitate the identification of targeted approaches for their effective inactivation.

In bulk-processed dairy products, preventing biofilm formation requires strict control of raw material contamination, frequent CIP procedures, short production cycles, control of temperature variations on surfaces, and hygienic engineering design of equipment and processes [54,67]. Nevertheless, additional control measures are often necessary to reduce the almost inevitable formation of thermophilic biofilms in industrial settings. These biofilms are resistant to conventional CIP procedures [85], prompting the development of alternative strategies to prevent or manage the adhesion of thermophilic biofilm-forming bacteria. Approaches include combined chemical and thermal treatments (e.g., 2% caustic solution and 1.8% nitric acid at 75 °C for 30 min [86]), the use of sanitizers such as hydrogen peroxide and peracetic acid following CIP procedures [46,87], application of hyperthermoacidic enzymes under heated acid conditions [88], cavitation treatments [89], and the use of bacteriophages or phage-derived endolysins specific to (*Para*)*Geobacillus* spp. [90,91]. Additionally, modifying the surface properties of stainless steel, such as altering metal ion composition of surfaces using polishers [92] or applying GRAS (Generally Recognized as Safe) bacteria to prevent colonization by other microbes [93], has shown potential to reduce the ability of mesophilic bacteria to adhere to processing equipment [94]. However, further studies are needed to evaluate these strategies for thermophilic spores. Reducing microfiltration pore size from 1.4 to 1.2 µm may enhance the removal of *G. stearothermophilus* spores from milk due to their tendency to form clusters [95].

Another important concern in controlling thermophilic spores is that culture-dependent enumeration methods often underestimate contamination levels [41,96]. Our recent findings indicate that, beyond the variations in eco-physiological requirements among thermophilic strains, limited germination and/or outgrowth to form visible colonies on rich nutrient plates are inherent characteristics of (*Para*)*Geobacillus* spp. [97]. Further research at the molecular and genetic levels is needed to better understand these limitations and to develop improved, standardized methods for accurately quantifying viable spores.

## 3. Food Safety Applications

One of the earliest applications of (*Para*)*Geobacillus* spores in ensuring food safety is controlling thermal sterilization processes. To verify sufficient treatment intensity for each batch, highly heat-resistant spores of *G. stearothermophilus* (D_121 °C_ = 1.3–5.4 min vs. 0.21 min for *Clostridium botulinum* spores) have traditionally been used as biological indicators to confirm sterility [14,41]. In addition, *G. stearothermophilus* spores can be used to ensure the effectiveness of hydrogen peroxide disinfectant treatments [15,98]. However, their use has decreased over time due to the development of cheaper chemical kits that do not need incubation time. Currently, *G. stearothermophilus* spores are employed in tests for detecting antibiotic residues in foods of animal origin [99,100]. Antibiotics are substances produced naturally, synthetically, or semi-synthetically by microorganisms, intended to kill or inhibit the growth of pathogens. Antibacterial antibiotics are primarily used therapeutically in livestock to treat diseases, requiring a withdrawal period—the time between the last dose of antibiotic given to animals and their slaughter or entry into the food chain—in order to prevent consumer exposure to hazardous residues [101,102]. Furthermore, the use of antibiotics as feed additives can prevent diseases and promote growth in healthy animals by improving nutrient absorption, reducing toxin formation, and decreasing immune system activity [102,103]. Health concerns over the massive use of antimicrobials in animals have led many countries, including the European Union (EU), and the USA, to ban the use of antibiotics for growth promotion [104,105,106]. However, it is still allowed in some countries, particularly in parts of Asia, Africa, and Latin America, though the trend is shifting toward more restrictions [107,108].

The main concern regarding the excessive and improper use of antibiotics in animals (e.g., failure to respect withdrawal periods or mishandling of animals) is the development of resistance and the transfer of resistant microorganisms to humans [102,109]. In addition, the ingestion of antibiotic residues through food can lead to organ toxicity, such as hepatic and reproductive damage, carcinogenicity, allergic reactions, and gastrointestinal dysbiosis in consumers [110]. The presence of antibiotic residues in raw materials also presents economic challenges for the fermented milk and meat industries by inhibiting the activity of beneficial microbes [101,111]. To mitigate these risks, regulatory bodies such as the EU [112] and Codex Alimentarius [113] have established maximum residue limits (MRLs) for authorized antibiotics in live animals and animal-derived products. Reliable detection methods are therefore required for routine control, as food containing residue concentrations that exceed the MRLs must be discarded.

In Europe, surveillance of antibiotic residues typically begins with rapid, low-cost screening tests based on microbiological methods or enzyme-linked immunosorbent assays (ELISA). Positive results must then be confirmed using validated analytical techniques [114]. Microbiological methods assess the presence of antibiotics through the inhibition of growth in sensitive bacterial strains [17,115], and are commonly used due to their speed, low cost, and ease of operation [17,86,115]. Immunological methods are sensitive and reliable but are generally designed to detect a single antibiotic or a small group of structurally similar compounds, which is a limitation considering that combinations of antibiotics are often used in practice [116]. Chromatographic techniques, mainly liquid chromatography coupled with tandem mass spectrometry (LC-MS/MS), are recognized as the gold standard for confirming the identity and concentration of positive samples due to their high precision, sensitivity, and specificity [117]. However, these methods require expensive equipment, sophisticated sample preparation, and trained personnel [118]. Furthermore, chromatographic methods may miss biologically active degradation metabolites of antibiotics that are not specifically targeted, but which can still be detected by microbiological methods and may pose a health risk to consumers [114].

Microbiological methods use one or more bacterial strains whose growth is inhibited at or below MRLs by a broad antibiotic spectrum, such as *E. coli* [18,119], *Bacillus* spp. [16,115,119], *Kocuria rhizophila* [119], and *G. stearothermophilus* [16,17,115]. These methods can be classified into Petri dish and test tube formats. Petri dish methods consist of several agar plates with different pH to selectively detect certain antibiotics and are inoculated with strains of varying antibiotic sensitivity. Results are interpreted based on inhibition zone diameters. The most widely used Petri dish method in Europe is the Four Plate Test (FPT), which includes three plates with *B. subtilis* spores at pH 6.0, 7.4, and 8.0, and one plate at pH 8.0 inoculated with *K. rhizophila* or *K. varians* [120,121]. Another common assay is Fast Antimicrobial Screen Test (FAST), a one-plate assay inoculated with *B. megaterium* spores [122]. Unfortunately, these methods are time-consuming (usually 18–24 h), labor-intensive, and require trained personnel, laboratory space, and high precision for measuring inhibition zones [17,115].

In recent years, test tube methods have increasingly replaced traditional plate methods due to their high throughput, portability, shorter detection time, ease of use, reliability, and comparable or superior sensitivity and specificity [17,86,115]. These methods consist of tubes or multi-well plates containing a solid nutrient medium, often supplemented with antibiotic-sensitizing agents, and the microbial sensor. Growth is detected through changes in pH, redox potential, or electrical properties of the medium, using appropriate indicators [115,119,123]. The chromogenic pH indicator bromocresol purple is widely used, remaining purple in antibiotic containing samples and turning yellow in non-contaminated ones. These tests allow for spectrophotometric monitoring of color changes, improving traceability, result accuracy, and sensitivity, while simplifying analysis compared to visual inspection [124]. Commercial test tubes usually use bacterial spores because of their higher resistance to adverse conditions and longer stability compared to vegetative cells [99]. *G. stearothermophilus* spores are widely used due to their high sensitivity to a broad spectrum of antibiotics, including β-lactams, tetracyclines, aminoglycosides, macrolides, sulfonamides, and lincosamides [17,18]. They exhibit rapid growth (detectable in less than 4 h), and their requirement for high incubation temperatures (55–65 °C) helps reduce the risk of contamination [99]. Several commercially available test tube kits use spores of different *G. stearothermophilus* strains to detect antibiotic residues in various food products, some of which are described in Table 1.

The most widespread application of commercially available test tube kits is the detection of antibiotics in milk, as these drugs are commonly used to treat mastitis. Many of them use *G. stearothermophilus* subsp. *calidolactis* strains, which are highly sensitive to β-lactams, though less sensitive to tetracyclines, sulfonamides, aminoglycosides, and quinolones [120]. A key advantage of milk-targeting tests is that samples can be directly added to the test tubes, with some exceptions [100,118]. In contrast, other food matrices such as eggs and other animal tissues often require sample preparation steps to eliminate interferences from matrix components such as heating of samples. Moreover, the complexity of animal tissues, along with their broad heterogeneity—such as differences in sample composition (blood vessels, organs, nerves, connective tissue, etc.), animal age, health status, and carcass variability—further complicate the development of reliable tests for these products [136]. Most of the commercially available tests (e.g., PremiTest, Explorer 2.0, Charm KIS) are designed for use on meat and/or kidney samples.

Despite the advantages and market availability of microbiological tube tests, improvements are still needed. First, ideal *G. stearothermophilus* spores should detect all major antibiotic families used in livestock—sulfonamides, fluoroquinolones, β-lactams, aminoglycosides, tetracyclines, phenicols, and oxazolidinones—at or below MRLs across all food matrices [101,102,123]. However, such performance requirements are not yet achievable with current tests [120]. Genetic engineering of suitable strains could enable the development of spore-based sensors with broader detection spectra and enhanced sensitivity. Second, test sensitivity is influenced by external factors such as the composition of the growth medium, the food matrix composition, and the spore concentration. For instance, calcium anions in milk improve the sensitivity to aminoglycosides but chelate tetracyclines reducing their bacteriostatic effect [118]. High somatic cell counts and natural inhibitors—especially in goat and sheep milk—increase the rate of false positive results [137,138]. Increasing the concentration of spores decreases detection time but lowers sensitivity and raises costs [118]. To minimize matrix interference and enhance sensitivity without laborious pretreatment steps unsuitable for on-site use [18,139], test media are often supplemented with additives. Examples include carboxymethylcellulose, fusidic acid, chloramphenicol (CAP), enrofloxacin, streptomycin, trimethoprim (TMP), 4-aminobenzoic acid, and phenylbutazone, which improve sensitivity to various antibiotic families [16,100,115,118,140]. Indeed, some commercial tests already incorporate one or more of these compounds—most commonly CAP, TMP, and/or phenylbutazone—to broaden the detection range of *G. stearothermophilus* spores [139,141].

## 4. Valorization of Agri-Food Residues

Food loss and food waste—here collectively referred to as agri-food residues—correspond to the reduction in food mass during production, post-harvest, and processing stages (food loss), or at the retail and consumer levels (food waste). Globally, it is estimated that one-third of all food produced is lost or wasted, contributing to food insecurity, undermining food sustainability, and causing serious economic and environmental impacts [142]. Plant-based biomass, including crop byproducts (bran, husks, bagasse, etc.), fruit and vegetable byproducts (leaves, seeds, peels, etc.), oilseed press cakes, and food waste (e.g., bread) represents one of the most abundant agri-food residues [143,144]. While a small proportion of these residues are repurposed for animal feed, agriculture fertilizers, and as raw materials for other industries, the majority are conventionally incinerated or disposed of in landfills causing health and environmental issues [145]. On the other hand, these residues are a source of compounds with potential health and economic benefits, obtainable through direct extraction or biomass transformation. In this context, biorefineries based on the microbial conversion of agri-food residues represent an economically and environmentally sustainable approach to produce a wide range of marketable compounds, such as biofuels, oligosaccharides, bioactive compounds, enzymes, bioplastics, among others [23,146,147,148,149,150,151,152], while simultaneously addressing the challenge of residue management. Thus, microbial biorefineries are emerging as key drivers in the transition toward a circular bioeconomy.

### 4.1. Advantages and Applications of (Para)Geobacillus spp. in Fermentation of Agri-Food Residues

For microbial biorefineries utilizing agri-food residues to be cost-effective, it is essential to employ microorganisms capable of degrading complex structures—such as lignocellulose and starch—through the production of a broad range of hydrolytic enzymes, as well as metabolizing a variety of mono- and oligosaccharides. Thermophilic microorganisms are especially attractive due to their faster growth and higher substrate conversion efficiency compared to mesophilic counterparts [153]. In addition, high-temperature fermentation offers several advantages, including reduced cooling costs, which are often required in large-scale fermentation involving mesophiles [154,155]. Notably, it has been estimated that increasing the process temperature by just 5 °C could lower production costs by more than $390,000 annually [154]. Additional benefits include reduced risk of contamination under non-sterile conditions, improved substrate solubility and mixing, easier maintenance of anaerobic conditions, and enhanced removal and recovery of volatile products [150,153,156,157,158,159]. Among the potential candidates, some (*Para*)*Geobacillus* spp. have shown promising traits for the valorization of agri-food residues [150,156,157,158,159].

The most investigated application of agri-food residues as fermentation feedstock for thermophilic microorganisms is the production of biofuels, especially bioethanol. Most bioethanol is currently produced using food crops (maize, sugar beet, and sugar cane), which are classified as first-generation (1G) biofuels. However, current production of these materials is insufficient to meet the global demand for biofuel while also satisfying the growing needs for human and animal food [160]. Agri-food residues, especially lignocellulosic or starchy biomass, offer a renewable, abundant, and non-edible alternative for producing second-generation (2G) biofuels [161]. The production of 2G bioethanol from lignocellulosic or starchy biomass comprises pretreatment, hydrolysis (or saccharification), and fermentation [161]. Pretreatment of lignocellulosic biomass—using physical, chemical, and/or biological methods—aims to break down lignocellulose, remove recalcitrant structures, and improve digestibility of cellulose and hemicellulose [161]. The pretreated biomass is then hydrolysed into simple sugars, preferably using a cocktail of cellulolytic enzymes. For starchy biomass, gelatinisation, liquefaction, and saccharification are performed at high temperatures combined with amylolytic enzymes [144]. Subsequently, fermentation is typically carried out by *Saccharomyces cerevisiae* due to its efficient conversion of sugars into ethanol and its tolerance to high product concentrations. Ethanol is then purified through additional processing steps [161].

One of the main cost barriers to 2G bioethanol production is the high price of exogenous enzymes required during the pretreatment and hydrolysis steps to efficiently release fermentable sugars [143]. A promising alternative to this drawback is the development of Consolidated Bioprocessing (CBP), a one-step process in which enzyme production, hydrolysis, and fermentation are carried out simultaneously by specialized microorganisms [162,163]. This approach promotes the use of thermophilic microorganisms capable of growing and producing cellulolytic and/or amylolytic enzymes that operate optimally within the same temperature range (50–60 °C), while also benefiting from the previously discussed advantages of high-temperature processes. Certain (*Para*)*Geobacillus* spp. strains, especially *P. thermoglucosidasius* strains, have shown desired traits for CBP [150,163], including hydrolytic enzyme production, utilization of diverse substrates (pentoses, hexoses, and short-chain polysaccharides), higher growth rates—even under microaerobic conditions—and greater ethanol tolerance compared to typical cellulolytic thermophiles such as *Clostridium thermocellum* [31,164]. Nonetheless, further improvements in both process design and strain development are necessary to optimize performance and make CBP commercially viable [165]. Table 2 compiles ethanol production (total ethanol generated, typically expressed in g/L or mM) and productivity (rate of ethanol formation over time, expressed in g/L/h) achieved during the fermentation of different agri-food residues by (*Para*)*Geobacillus* spp.

The development of genetic engineering tools for *P. thermoglucosidasius* has enabled the creation of strains with enhanced ethanol production [153,167,168]. The most successful example to date is *P. thermoglucosidasius* TM242, which was metabolic engineered from the type of strain (NCIMB 11955) to enhance ethanol production (Table 2). This was achieved by deleting the genes encoding lactate dehydrogenase and pyruvate formate lyase and up-regulating the gene encoding pyruvate dehydrogenase (Δ*ldh* Δ*pfl pdh*^up^ genotype) [169,170]. As a result, bioethanol production from palm kernel cake (PKC) hydrolysate by the strain TM242 was 4.7-fold higher than that achieved with *S. cerevisiae*, while requiring milder pretreatment conditions and lower enzyme loadings [151]. The strain TM242 was patented by TMO Renewables Ltd. (London, UK); however, attempts to replicate the Δ*ldh* Δ*pfl pdh*^up^ genotype in the strain LS242, resulted in 7.6-fold lower ethanol production in glucose than TM242, likely due to spontaneous mutations during strain development [171]. To further improve production, LS242 was engineered to express recombinant thermostable enzymes capable of efficient cellulose degradation. More specifically, the strains *P. thermoglucosidasius* BZ243 and BZ244 were constructed by chromosomal integration of a β-1,4-glucosidase gene from *Thermoanaerobacter brockii*, combined with different plasmid-borne cellulolytic enzymes from *Clostridium thermocellum*, *Thermobifida fusca*, and *Caldicellulosiruptor bescii*. Using wheat straw as a substrate with a simple pretreatment with nitric acid and ammonia, strains BZ243 and BZ244 achieved ethanol productions of 3.9 and 3.4 g/L, respectively, corresponding to 1.6- and 2.0-fold increases compared to LS242 [150] (Table 2).

Another approach to enhancing ethanol production is the successive or simultaneous co-culturing of (*Para*)*Geobacillus* spp. strains with other microorganisms. For example, the sequential incubation of *P. thermoglucosidasius* ATCC 43742, followed by the highly ethanol-tolerant *Thermoanaerobacter ethanolicus* ATCC 31938 to exploit remaining substrates in non-pretreated food waste from a cafeteria, resulted in the production of 18.4 g/L of ethanol (Table 2), corresponding to 105.8 L of ethanol per US ton of food waste [166]. The substrate mass recovery rate—defined as the proportion of sugars accounted for as known products at the end of the process—was 92%, and the remaining waste was suitable for methane production [166]. A similar mass recovery, but with lower ethanol production (3.72 g/L), was achieved from the digestion of corn stover by *Geobacillus* sp. DUSELR13 leveraging its thermostable xylanases and cellulases, followed by fermentation with *P. thermoglucosidasius* ATCC 43742 (Table 2) [152]. Ethanol production could potentially be improved using a *P. thermoglucosidasius* Δ*ldh* Δ*pfl pdh*^up^ strain. In another example, simultaneous fermentation of bean curd refuse—a by-product with an annual production of approximately 700,000 tons in Japan—by a cellulolytic aerobic *Geobacillus* (kpuB3) and a hemicellulolytic anaerobic *Thermoanaerobacterium* (kpu04), both isolated from compost, synergistically enhanced ethanol production, reaching 1.24 g/L [163] (Table 2).

Comparing ethanol production performance across studies is challenging due to differences in the composition of the agri-food residues, the (*Para*)*Geobacillus* spp. strains used (whether alone or in co-culture), and variations in fermentation conditions. Nevertheless, it can be inferred from Table 2 that the highest ethanol titers were achieved by the co-culture of *P. thermoglucosidasius* ATCC 43742 and *T. ethanolicus* ATCC 31938 using a complex substrate such as cafeteria food waste. While co-culture systems—particularly when implemented sequentially—can increase ethanol titers and tolerance range, they often prolong the overall fermentation time and reduce overall productivity. In this context, the use of genetically engineered strains may offer a more efficient and economically viable alternative on simpler, more accessible substrates. For instance, although cafeteria food waste supported higher ethanol titers than PKC hydrolysate (Table 2), the ethanol conversion efficiency was 2.0-fold higher with the latter, achieving a theoretical yield efficiency of 92% (i.e., actual ethanol produced relative to the maximum theoretical stoichiometric yield from the substrate) [151]. This highlights that, despite the advantages of co-cultures in handling complex substrates, fermentation efficiency can be maximized by coupling genetic modifications once the substrate composition has been simplified. However, the large-scale application of *P. thermoglucosidasius* for the utilization of agri-food residues predominantly composed of complex lignocellulosic or structurally heterogeneous materials—such as wheat straw, corn stover, and bean curd—remains a significant challenge, even when employing co-cultures or engineered strains with enhanced cellulolytic activity (e.g., BZ243 and BZ244; Table 2). Achieving competitive yields from these feedstocks may require the exploitation of Simultaneous Saccharification and Fermentation (SSF) [144], which involves the external addition of cellulolytic enzymes during fermentation, although the process can be cost-prohibitive compared to CBP [165]. A notable example is the genetically modified strain *P. thermoglucosidasius* TM333—engineered from TM242 to overexpress a second amylase gene from *G. stearothermophilus* [153,172,173]—combined with a crude α-amylase extract from the same strain for waste bread fermentation. This approach resulted in higher ethanol production (14.2 g/L) and theoretical yield efficiencies (94–96%) compared to *S. cerevisiae* (3.7 g/L and 27%, respectively) [144].

Unfortunately, although both CBP and SSF strategies have demonstrated the potential to exceed 90% theoretical ethanol yield [144,151]—a critical threshold for industrial feasibility—volumetric ethanol concentrations typically remain below the economic benchmark of 5% *v*/*v* [174]. This limitation is likely due to the ethanol toxicity threshold of *P. thermoglucosidasius* TM242 (>15.78 g/L, 2% *v*/*v*), which restricts both cell growth and ethanol accumulation [165]. As a result, final titers typically fall below 2% *v*/*v*, thereby limiting substrate loading and rendering the process economically unviable [144,174]. To overcome this bottleneck, hot air gas stripping has recently been proposed as a promising technique for continuous ethanol removal during fermentation, allowing titers to surpass the 5% *v*/*v* threshold [175]. However further optimization is required, as up to 49% of the ethanol is lost during the stripping process [175]. In parallel, an evolved strain with enhanced ethanol tolerance (>26.5 g/L, 3.4% *v*/*v*) has recently been developed [174], which could serve as an alternative or complement to in situ product removal strategies to alleviate ethanol toxicity and thus enhance ethanol production from complex agri-food residual substrates. Regarding other biofuels, *P. thermoglucosidasius* KCTC 33548 has also shown potential for hydrogen production using potato peel as a substrate, although the yields remain economically unviable [176]. In addition, *P. thermoglucosidasius* strains have been engineered for the production of butanol derivatives [177], but these strains have not yet been applied to the valorization of agri-food residues. Beyond fermentation, *(Para)Geobacillus* spp. are capable of producing thermostable lipases, which can be exploited exogenously for the transesterification of fatty acid-rich residues (e.g., used cooking oil, animal fat) into 2G biodiesel [157,158,159]. Thermolipases offer advantages such as high stability and mass transfer rates compared to commercially available mesophilic lipases [158]; however, further research is needed to fully harness their potential as biocatalysts in industrial applications.

Another important high-value product that can be obtained through fermentation of lignocellulosic biomass by *P. thermoglucosidasius* is riboflavin [178]. Riboflavin is an essential component of cellular metabolism and is widely used as a food additive and dietary supplement [178]. Currently, commercially available riboflavin is produced via fermentation using mesophilic bacteria. To reduce fermenter cooling costs, *P. thermoglucosidasius* DSM 2542 was engineered to incorporate a gene cluster encoding riboflavin synthesis (the *rib* cluster from *G. thermodenitrificans* NG80-2). Additional modifications to the biosynthetic pathway using different xylose-inducible promoters enabled the selective utilization of xylose for riboflavin production and glucose for growth, resulting in a strain capable of producing riboflavin at 121.0 mg/L from a fermentation medium containing 0.5% corn cob hydrolysate [178]. However, these titers remain significantly lower than those achieved with mesophilic microorganisms such as *B. subtilis* (>10 g/L) [179]. Therefore, further metabolic and process optimizations are required to enhance riboflavin production in *P. thermoglucosidasius*.

Starchy residues offer a cost-effective and sustainable alternative to crops for microbial lactic acid production. Lactic acid is widely used as a food additive and in the chemical synthesis of biodegradable, food-grade bioplastics [149,180]. *G. stearothermophilus* is a promising candidate due to its ability to convert starch directly into lactic acid, bypassing the glucose extraction step required by most lactic acid-producing microorganisms and thus reducing production costs [149]. For instance, *G. stearothermophilus* DSM 494 produced 59 g/L of optically pure (98%) L-lactic acid in 48 h from potato residues [181], while other strains reached 5.3 g/L of lactic acid in 24 h from rice waste [149]. However, further improvements are needed before lactic acid production can be scaled up for industrial use, including strain engineering to minimize co-metabolite formation, implementation in continuous membrane bioreactors, and optimization of fermentation conditions (temperature, pH, shaking, carbon and nitrogen sources, etc.) [149,181]. Promising advances have already been achieved through a combined metabolic engineering and adaptive evolution approach, yielding a *P. thermoglucosidasius* strain capable of producing 151.1 g/L of lactic acid from 100 g/L of glucose after 48 h, with a productivity of 3.1 g/L/h [182]. However, its performance using food waste as feedstock remains to be evaluated.

Other reported cases of agri-food residue valorization through fermentation with (*Para*)*Geobacillus* spp. include terpene production from waste bread. Terpenes are natural compounds with diverse industrial applications, particularly the flavor industry. Styles et al. [183] engineered a *P. thermoglucosidasius* strain carrying a heterologous mevalonate pathway derived from *Saccharolobus solfataricus* and *G. stearothermophilus*, along with a thermostable terpene synthase from *Roseiflexus* sp. Rs-1. This strain produced terpenes from bread waste at titers (14 mg/L) comparable to those achieved in early studies using *E. coli* in Luria–Bertani (LB) broth [184]. However, to be economically feasible, higher titers (>20 g/L) have been achieved over time using mesophilic microorganisms (e.g., *E. coli* or *S. cerevisiae*) in complex laboratory media [185]. Therefore, further research is needed to improve terpene yields in *P. thermoglucosidasius* [183]. In addition, (*Para*)*Geobacillus* spp. can utilize crops residues to produce thermophilic enzymes such as proteases and xylanases [23,146,147], as well as xylooligosaccharides [23,147], which hold great potential in the food industry (see below). Beyond plant-based residues, animal-derived waste streams such as feather waste—generated in large quantities worldwide—also represent valuable feedstocks. *G. thermodenitrificans* PS41 has demonstrated the ability to convert feather waste into bioactive compounds with antibacterial and anticancer activity [148], as well as into a biofertilizer substance [186], although process optimization is still required to enhance economic feasibility.

Another way to valorize agri-food residues is through compost production, which provides a valuable source of nutrients for plants. Various plant residues—maize straw [187], spent coffee grounds [188], rice straw [189], sugar cane leaves [190], and burnt coffee grounds [191]—can be composted alone or in combination with animal refuses [187,190]. The process comprises four stages, during which mesophilic and thermophilic organisms decompose the organic matter into a stable, humus-like substance [191]. The thermophilic or “active” phase (45–70 °C) is characterized by rapid decomposition, predominantly driven by *Bacillus* and *Geobacillus* spp. [187,191]. Inoculation with thermophilic microorganisms has numerous benefits: it prolongs the thermophilic phase by rising the temperature, pH, and germination index; enhances the efficiency and speed of biodegradation; increases the content of aromatic compounds and humification; and helps preserve nitrogen in compost [187,190,191]. Some (*Para*)*Geobacillus* spp. strains are particularly important in this process due to their ability to decompose lignin at high temperatures, a process that is typically carried out by fungi but is inhibited under thermophilic conditions [191]. Additionally, they produce amylases, endoglucanases, cellulases, carboxymethyl cellulases, xylan and gelatin hydrolases, endoxylanases, β-xylosidases, and α-arabinofuranosidases, among other enzymes for further biomass degradation [189,191,192,193,194]. Even so, co-culture with other bacteria (e.g., *Bacillus*, *Ureibacillus*, *Thermobacillus*, *Paenibacillus*) is required in order to achieve maximum benefits [188,190,191].

### 4.2. Limitations of (Para)Geobacillus spp. in Fermentation Processes

Most advancements in fermentation have focused on *P. thermoglucosidasius*, as it is the most studied (*Para*)*Geobacillus* species for genetic manipulation [165,195]. However, knowledge of the physiology and metabolism of species across both genera remains limited. Despite its advantages, *P. thermoglucosidasius* faces significant challenges in maintaining high and viable biomass yield, especially in fermentation processes [196,197]. Extensive cell death has been observed not only during the stationary phase but also during exponential growth in *P. thermoglucosidasius* and other *Geobacillus* spp., such as *G. thermoleovorans* [182,196,197]. For instance, up to 30% of the *P. thermoglucosidasius* population may undergo sudden cell death during exponential growth [197]. As a consequence, cell death forces the need for repeated reinoculation of the fermentation medium to maintain high production yields [166].

Understanding the cellular and molecular mechanisms underlying cell lysis in (*Para*)*Geobacillus* spp. requires further investigation to mitigate this phenomenon and increase profitability. It has been suggested that cell lysis may be a genetically programmed differentiation process, although environmental conditions such as temperature, medium composition, and aeration may also influence its occurrence [196]. Zhou et al. [198] observed that supplementing the fermentation medium with acetic acid—as an alternative source of acetyl-CoA—partially restored growth of a *P. thermoglucosidasius* Δ*ldh* Δ*pfl* strain in minimal media containing acetic acid and either glucose or cellobiose for ethanol production. As this intervention was insufficient to sustain robust growth, adaptive evolution was performed to select strains with increased biomass and ethanol production. The evolved strains commonly acquired loss-of-function mutations in the *aprt* gene, which was predicted to encode adenine phosphoribosyltransferase—an enzyme involved in the purine nucleotide salvage pathway—as well as in the *spoIIIAA* gene, which encodes stage III sporulation protein AA, along with four to five additional SNPs. Notably, a Δ*ldh* Δ*pfl* Δ*aprt* Δ*spoIIIAA* strain exhibited increased sugar and acetate consumption and enhanced ethanol production than the parental strain, although to a lesser extent than the evolved strain. The synthetic mutant also showed increased biomass, but this improvement was better maintained over time in the evolved strain as well [198].

Previous knowledge of factors involved in cell lysis in *Bacillus* spp. could help mitigate the limitations posed by cell death in industrial applications of (*Para*)*Geobacillus* spp. In *Bacillus* spp., cannibalism toxins, toxin-antitoxin systems, and peptidoglycan hydrolases may contribute to cell lysis [199,200,201,202,203,204,205,206], and deletion of genes from these categories has led to improved recombinant protein production [206]. However, Liu et al. [182] explored, albeit unsuccessfully, whether deleting two toxin-antitoxin systems—identified by genome mining in a *P. thermoglucosidasius* DSM 2542 strain engineered for lactic acid production—could alleviate cell lysis. To improve strain performance, adaptive evolution was employed, resulting in increased cell density and enhanced lactic acid production. The evolved strains carried multiple mutations in genes related to proteolysis, stress response, and transcriptional regulation, all of which may contribute to cell lysis [182]. Therefore, further research into the genetic and regulatory mechanisms underlying sudden cell death in thermophilic bacteria is essential to enable the industrial-scale production of valuable compounds.

## 5. Obtention of Thermostable Enzymes for Food Applications

Government regulations promoting eco-friendly products and processes in regions such as the US and EU have driven growing demand for enzymes as sustainable alternatives to chemical catalysts across several industries [207], including pharmaceuticals, detergents, textiles, leather, paper, medicine, biofuels, bioremediation, and food and beverages [208,209,210]. The global enzyme market, led by North America and Europe, is steadily expanding, with the food and feed industries together accounting for approximately 55–60% of total enzyme consumption [208]. This review focuses specifically on enzyme applications within the food industry. The increasing use of enzymes in food production has led to more stringent regulatory measures in both the EU and the US. In the US, enzymes are classified as food additives, whereas the EU is currently establishing an official list of approved enzymes [209].

Enzymes have traditionally been extracted from plants and animals; however, recent advances in biotechnology have sharply increased enzyme production from microorganisms, owing to their ease of production and manipulation. In addition, microorganisms can be exploited to produce stable and functional enzymes at high temperatures (referred to as thermophilic enzymes or thermoenzymes), which offer several advantages over their mesophilic counterparts, including reduced hydrolysis time, lower risk of microbial contamination, and increased solubility of substrates and products [210]. Furthermore, thermoenzymes often exhibit tolerance to other harsh industrial conditions, such as high pressure, extreme pH, and denaturing solvents [210].

Due to their ecological requirements, (*Para*)*Geobacillus* spp. strains are natural sources of a wide variety of enzymes stable at temperatures between 50 and 75 °C and at pH values of 5–9 [211]. However, as is common with many thermophilic microorganisms, (*Para*)*Geobacillus* spp. produce low biomass yields. This limitation necessitates the use of recombinant production systems, primarily involving the cloning of thermoenzyme genes into a faster-growing and easier-to-handle host, to meet industrial requirements for efficiency and profitability [210]. Alternatively, thermoenzymes can be obtained through the directed evolution of mesophilic enzymes or synthetic protein engineering strategies, such as error-prone polymerase chain reaction (PCR) or computer-assisted enzyme engineering strategies. However, successful outcomes require advanced genetic engineering techniques and a deep understanding of enzyme structure [210,212,213]. The most important thermoenzymes from (*Para*)*Geobacillus* spp. with potential uses in various food industry processes are described below. Enzymes that are commercially available or have demonstrated benefits in food-related applications are summarized in Table 3.

### 5.1. Amylases

Amylases catalyze the hydrolysis of starch bonds and other related polysaccharides and oligosaccharides, resulting in progressively smaller dextrins and simple sugars such as glucose and maltose [222,223]. The amylase family comprises endoamylases, exoamylases, transferases, and debranching enzymes, with α-amylases (endoamylases) and pullulanases (debranching enzymes) being the most efficient starch-hydrolyzing enzymes [224].

In the food industry, thermostable amylases are generally preferred, as key starch processing steps generally occur at high temperatures [225]. Thermostable amylases play a crucial role in the production of sweeteners—such as glucose, fructose, maltodextrins, and syrups. During the liquefaction stage, these enzymes break down starch into small polysaccharides and oligosaccharides at high temperatures (80–90 °C), reducing viscosity and enhancing fluidity [222]. In addition, they are used in the saccharification step, typically at 50 °C or higher temperatures and near-neutral pH, to produce maltose and glucose while minimizing browning reactions [226]. In baking, amylases are commonly added to reduce dough viscosity, enhance bread quality—including volume, texture, taste, color, shelf-life, and toasting properties—and delay crumb firming during storage through their anti-staling effects [216,227,228,229]. In juice, the presence of starch and other polysaccharides—such as hemicellulose, cellulose, and pectin—contributes to turbidity and viscosity, which is undesirable in certain products from the consumer’s perspective [230]. Therefore, juice production often involves a clarification step at 40–60 °C with an enzymatic cocktail that includes amylases [230,231]. Additionally, thermostable amylases enable starch gelatinization during mashing in brewing [232].

Many strains of (*Para*)*Geobacillus* spp. produce thermostable α-amylases, such as *G. stearothermophilus* JT2 [226], *Geobacillus* sp. LH18 [19], *G. thermodenitrificans* HRO10 [20], *G. stearothermophilus* [216,228], *Geobacillus* sp. IIPTN [233], *G. thermoleovorans* KNG 112 [234], and *Geobacillus* sp. GS33 [235], among others. Notably, the α-amylase of *Geobacillus* sp. IIPTN can work efficiently even at 120 °C [233]. Furthermore, the α-amylases produced by *Geobacillus* sp. LH18 and *G. thermodenitrificans* HRO10 exhibit both thermostability and resistance to phytic acid—a common phosphate storage compound in plant tissues—making them valuable for corn processing [19,20]. *Geobacillus* sp. DS3 produces a thermostable α-amylase capable of generating porous starch, which is known for their adsorbent and encapsulant properties, making it suitable as a flavor carrier in the food industry [22].

Beyond α-amylases, other starch-hydrolyzing enzymes have been isolated from (*Para*)*Geobacillus* spp. For instance, a pullulanase from *G. stearothermophilus* ADM-11 has recently shown promising results for food industry applications [236], and amylopullulanases from *G. thermoleovorans* have been utilized in starch liquefaction and saccharification to obtain maltose, maltotriose, and maltotetraose syrups [217]. A thermostable 1,4-α-glucan branching enzyme produced by *G. thermodenitrificans* has been approved by the European Food Safety Authority (EFSA) for use in cereal-based dough preparation to reduce staling and extend shelf-life, as well as in rice or pasta processing prior to cooking to delay retrogradation [21]. Cyclodextrin glycosyltransferases isolated from different (*Para*)*Geobacillus* strains—*P. thermoglucosidasius* CHB1, *G. stearothermophilus* ET1, and *G. stearothermophilus* NO2—can be employed for the production of cyclodextrins [237,238,239]. These food additives are used to encapsulate and protect small molecules such as vitamins, flavors, colorants, and unsaturated fats from degradation, oxidation, and loss through volatility or sublimation, and to improve the taste of non-caloric sweeteners [237,238,239].

### 5.2. Xylanases

Xylan is one of the most abundant hemicellulosic compounds. This heteropolysaccharide has a complex structure that requires different xylanolytic enzymes for its complete hydrolysis [218,219]. Thermostable xylanases are often included in enzymatic cocktails used for juice clarification. The addition of xylanases produced by (*Para*)*Geobacillus* spp. (*P. galactosidasius* BS61, *G. vulcani* GS90, and *Geobacillus* sp. TF16) for clarification of various juices—such as orange, pomegranate, apricot, peach, apple, grape, and kiwi—has been reported to decrease their turbidity while increasing the yield of reducing sugars [218,219,220].

Other applications of xylanases include the baking industry, where they are used to reduce bread staling and stickiness, extend shelf-life, increase rise rates and final volume in dough, pastry, and bread, and improve crumb structure [240]. The use of immobilized recombinant xylanase from *Geobacillus* sp. TF16 enabled shorter processing times and reduced enzyme quantities to enhance the rise rate of dough and pastry, compared to xylanase from *B. licheniformis* P11(C) [218]. Moreover, xylanases from *G. stearothermophilus* T6, *G. thermodenitrificans* A33, and *G. thermodenitrificans* TSAA1 can also be exploited to produce xylooligosaccharides and arabinoxylooligosaccharides—prebiotic ingredients used in food and nutritional supplements—from lignocellulosic biomass [23,24,25].

### 5.3. L-Arabinose Isomerase

L-arabinose isomerase (L-AI) is an aldo-keto isomerase of great importance in the synthetic production of rare sugars, such as L-ribulose (via the isomerization of L-arabinose) and D-tagatose (via the isomerization of D-galactose) [241]. D-tagatose is a non-caloric sweetener with 92% of the sweetness of sucrose [241,242] and provides health benefits, including the reduction in symptoms associated with anemia, hyperglycemia, and diabetes [242,243]. It is recognized as GRAS by the U.S. FDA (Food and Drug Administration) [244], and its use is approved as a novel food in the EU [245]. Its chemical synthesis is not environmentally friendly, requiring high pressures and high temperatures along with complex purification steps [243,246]. Therefore, the use of thermostable L-AI as a biocatalyst represents an attractive alternative, as high temperatures (50–70 °C) enhance the conversion rate to tagatose [31,242,247]. L-AIs have been isolated from several strains of (*Para*)*Geobacillus* spp. *G. stearothermophilus* GSAI [248], *G. thermodenitrificans* GTAI [249], *P. thermoglucosidasius* KCTC 1828 [250], *G. stearothermophilus* US100 [251], *G. stearothermophilus* KCCM 12265 [252], *G. stearothermophilus* IAM 11001 [253], and *G. stearothermophilus* DSM 22 [254]. In addition, the development of genetic engineering tools has allowed the creation of (*Para*)*Geobacillus* L-AIs with improved tagatose production [249]. For instance, a triple site-directed variant enzyme (F280N) from *G. thermodenitrificans* GTAI has shown the highest reported productivity for D-galactose isomerization (4.92 g/L/h) [249]. Its expression in permeabilized and immobilized cells of *Corynebacterium glutamicum* (a GRAS host) increased productivity to 165 g/L, resulting in a 2-fold increase compared to *B. subtilis* expressing L-AI from *Lactobacillus fermentum* [255]. The thermostability and catalysis of some enzymes produced by (*Para*)*Geobacillus*, including L-AIs, usually need the presence of metal ions, such as Mn^2+^ or Co^2+^. However, the use of Co^2+^ is not allowed in the production of D-tagatose for food applications due to its toxicity [241,254]. Consequently, some studies have aimed to develop metal-independent thermophilic enzymes. Although the presence of metallic ions is essential to enhance the conversion of D-tagatose, certain mutations have enabled high production rates in their absence [252,255].

Other studies have focused on engineering enzymes to increase their activity under more acidic conditions such as pH 6, since alkaline pH combined with high temperatures favors the Maillard reaction [256,257]. Additionally, the simultaneous application of multiple recombinant enzymes—such as β-galactosidase and L-AI from *G. stearothermophilus*, along with D-glucose isomerase and D-allulose epimerase from other microorganisms—has been proposed as a strategy to produce various rare sugars while reducing production costs [258]. However, further research is needed to ensure its profitability.

### 5.4. β-Galactosidases

β-Galactosidases are important enzymes in the dairy industry used to hydrolyze lactose into glucose and galactose, resulting in low-lactose or lactose-free milk products [259]. Nowadays, lactose hydrolysis can be performed under refrigerated conditions by adding the enzyme directly to the storage tank for 24 h, or after UHT treatment under aseptic conditions, where a sterile enzyme is introduced before packaging and milk is stored at room temperature for 3 days. Since lactose-free and low-lactose UHT milk is very susceptible to the Maillard reaction due to the high content in reducing sugars and proteins, the enzyme is usually added after the heat treatment [260]

β-Galactosidases capable of withstanding long-duration and low-temperature pasteurization treatments (62.8–65.6 °C, 30 min) offer the potential for in situ lactose hydrolysis during thermal treatment, thus reducing production time and allowing earlier product release to market [26]. β-Galactosidases from *G. stearothermophilus* can function at such temperatures [261]. However, the use of mesophilic enzymes is still predominant in the dairy industry. Thermostable β-galactosidases are also interesting for producing galacto-oligosaccharides [27]—prebiotics associated with various health benefits, including modulation of gut microbiota, enhancement of skin health and immune function, improved calcium absorption, and prevention of constipation, among others [27,262].

### 5.5. Lipases

Triglycerides are hydrolyzed to glycerol and free fatty acids by lipases. The lipases obtained from the genus (*Para*)*Geobacillus* have potential for industrial uses such as the production of flavoring additives, the enhancement of lipid digestibility, and the conversion of oils and fats into high-value products [28,29]. For instance, a lipase from *G. thermocatenulatus* BTL2 has been shown to hydrolyze rapeseed oil, increasing omega-3 fatty acid content [30]. A recombinant lipase from *G. stearothermophilus* L1 can hydrolyze solid lipids such as palm oil and beef tallow, which are major sources for the production of fatty acids in the food industry [263]. This type of hydrolysis is nearly impossible to achieve using lipases from mesophilic microorganisms [264].

### 5.6. Proteases

Proteases are enzymes that catalyze the hydrolysis of proteins into short peptides and amino acids. (*Para*)*Geobacillus* spp. proteases have been extensively studied for their applications in the detergent industry [265], but they also show promise in the food and feed industry. For instance, thermoproteases from (*Para*)*Geobacillus* spp. can be used in the production of aspartame precursors. Aspartame, a methyl ester of aspartic acid and phenylalanine, is an artificial sweetener widely used as a food additive. There is a commercially available metalloendopeptidase derived from *G. stearothermophilus*, termed Thermolysin (Sigma-Aldrich, St. Louis, MO, USA), used for the synthesis of aspartame precursors [31]. The strain *G. thermopakistaniensis* MAS1 produces a thermostable aspartate aminotransferase (AST), which can be employed for the production of amino acids such as aspartic acid [266].

Soybean is an inexpensive protein source for both food and feed, and its fermentation by *G. stearothermophilus* enhances its nutritional value and bioactivity. The proteases secreted by *G. stearothermophilus* increase the value of crude and soluble proteins and peptides, while enhancing antioxidant activity and angiotensin-converting enzyme (ACE) inhibitory activity [32]. Furthermore, the fact that the process is performed at high temperatures under non-sterile conditions reduces production costs [32]. A recombinant protein from *G. stearothermophilus* CAU209 has also been shown to produce ACE-inhibitory peptides from whey protein hydrolysate, making it suitable for the production of antihypertensive hydrolysates and peptides, with potential application in the production of partially hydrolyzed formulas [221].

### 5.7. L-Asparaginases

L-asparaginase is an enzyme that hydrolyzes L-asparagine into aspartic acid and ammonia [267]. Although L-asparaginase is highly valuable in the medical industry, it can also be used in the food industry to mitigate the formation of the carcinogenic acrylamide. Acrylamide forms during the Maillard reaction when the α-amino group of free L-asparagine reacts with the carboxyl group of reducing sugars [268]. Its formation can be controlled by limiting the temperature or duration of the heating process, lowering pH, controlling storage conditions [268,269,270], and/or reducing the levels of sugars or L-asparagine [271]. However, these approaches may negatively affect the organoleptic properties of food. For this reason, the use of L-asparaginase is considered a promising solution to reduce acrylamide content [271,272,273]. L-asparaginase has been isolated from various *Geobacillus* spp. (*G. kaustophilus*, *G. thermopakistaniensis*, and *G. thermodenitrificans*) [271,273,274,275], although only a recombinant enzyme from *G. kaustophilus* DSM 7263 has so far been evaluated in starch food models with very encouraging results [271,273].

## 6. Conclusions

(*Para*)*Geobacillus* spp. hold considerable importance in the industrial production of shelf-stable, heat-treated products, as their spores and/or enzymes can lead to spoilage and economic losses. Their occurrence in these products is associated with inadequate hygiene and processing conditions, especially in bulk-heat-treated products where insufficient cleaning procedures may promote biofilm formation. Although their detection is often primarily a matter of poor hygiene—given their obligate thermophilic nature—the risk of spoilage is expected to increase as climate change intensifies. Consequently, several strategies are being explored to reduce the impact of (*Para*)*Geobacillus* spp. in food environments, including the optimization of CIP procedures, the use of alternative surface materials to limit biofilm formation, and the development of novel spore eradication methods that avoid excessive thermal processing. Further research on the molecular mechanisms of (*Para*)*Geobacillus* spp. spore formation and resistance, as well as the microbial and physicochemical composition, structure, and dynamics of thermophilic biofilms, is essential for designing effective control and prevention methods.

On the other hand, these bacteria have attracted the food industry’s interest due to their numerous beneficial applications, some of which may still be undiscovered. Notably, *G. stearothermophilus* spores are used as biological indicators in rapid screening tests to detect antibiotic residues in various foods. While commercially available tests are excellent for detecting certain antibiotic groups in specific products, there is a current need to enhance their sensitivity to a wider array of antibiotics and to levels below regulatory maximum residue limits. This could be achieved by synthetically modifying the intrinsic sensitivity of the reporter strain or by modulating environmental factors affecting sensitivity, such as the composition of the growth medium and the pre-treatment of samples.

(*Para*)*Geobacillus* spp. are promising candidates for fermenting agri-food residues to produce high-value compounds like biofuels, compost, food ingredients, and technological coadjutants, thus contributing to the sustainability and circular economy of food production systems. Their main advantages stem not only from their thermophilic trait—which includes increased production rate, reduced cooling costs, and a low risk of contamination—but also from their capability to produce hydrolytic enzymes that degrade lignocellulosic and starchy materials in fermentable sugars, thereby reducing the need of exogenous enzyme addition. However further improvements are needed to make the process economically viable, which begin with exploring the genetic diversity within both genera, gaining a deeper knowledge of their metabolic pathways, further developing genetic engineering techniques, understanding ecophysiological requirements and intrinsic cell autolysis, and optimizing co-culture strategies.

Finally, (*Para*)*Geobacillus* spp. are also a source of several thermostable enzymes with many applications in food processing. However, most of them have not yet been commercially implemented. Therefore, their characteristics should continue to be investigated, and the production of recombinant enzymes optimized, especially as new trends demand novel applications for these enzymes in the food industry.

## Figures and Tables

**Table 1:** Commercial tests and research assays for detecting antibiotic residues in food using *G. stearothermophilus* spores as biological indicators. MRL stands for Maximum Residue Limit, which is defined as the highest legally permitted level of antibiotic residue in food within a given region.

Commercial Test	Microorganism	Target Foods	Antibiotics Detection	References
BRT AiM, BR-Test, BR-AS, BR-Blue Star (AiM-Analytik in Milch Produktions-und Vertriebs GmbH, München, Germany)	*G. stearothermophilus calidolactis* C953	Raw cow, sheep, and goat milk	Mainly β-lactams (penicillin and cephalosporin) at or below EU MRLs. Aminoglycosides, macrolides, sulfonamides, tetracyclines, and chloramphenicol above- EU MRLs.	[125,126]
Copan milk test (Copan Italia SpA, Brescia, Italy)	*G. stearothermophilus calidolactis* *	Raw, heat-treated, and powdered milk from cow, sheep, and goat	β-lactams and sulfonamides at or below EU MRLs. Tetracyclines, aminoglycosides, macrolides, and others above EU MRLs	[127]
Delvotest SP-NT and Delvotest T (DSM Food Specialties, Delf, The Netherlands)	*G. stearothermophilus calidolactis* *	Cow, sheep, buffalo, and goat milk and milk products	40–65 antibiotics (β-lactams, tetracyclines, sulfonamides, macrolides, glycopeptides, aminoglycosides, and others) at or above EU MRLs	[128,129]
Eclipse FARM 3G (ZEU-Inmunotec SL, Zaragoza, Spain)	*G. stearothermophilus calidolactis* *	Raw, heat-treated, skim milk, and powdered milk from cow, sheep, goat, and buffalo	More than 50 antibiotics of 8 groups (β-lactams, tetracyclines, sulfonamides, macrolides, aminoglycosides, lincosamides, anasamycins, and sulfones) at or below EU MRLs	[130]
Eclipse FARM 4G (ZEU-Inmunotec SL, Zaragoza, Spain)		Raw milk		[130]
Charm Blue-Yellow II (Charm Sciences, Lawrence, MA, USA)	*G. stearothermophilus calidolactis* *	Raw and ultra-pasteurized cow milk. Goat and sheep milk using longer incubation times	29 antibiotics (β-lactams, sulfonamides, aminoglycosides, and specially tetracyclines) at or below EU MRLs	[131]
Charm Cowside II (Charm Sciences, Lawrence, MA, USA)	*G. stearothermophilus calidolactis* *	Raw commingled and ultra-pasteurized cow milk.	11 antibiotics (β-lactams, sulfonamides, tetracyclines, macrolides, and aminoglycosides) at or below US MRLs and 30 antibiotics (β-lactams, sulfonamides, tetracyclines, macrolides, and aminoglycosides) at or below EU MRLs	[131]
PremiTest (R-Biopharm AG, Darmstadt, Alemania)	*G. stearothermophilus calidolactis* *	Eggs, meat (beef, pork, chicken), fish, shrimps, feed, kidney, and liver	β-lactams, cephalosporines, macrolides, tetracyclines, sulfonamides, aminoglycosides, quinolones, amphenicols, and polypeptides for beef, pork, and poultry at or below EU MRLs. Fish, shrimps, eggs, kidney, liver, and feed matrices require customer validation	[122,132,133]
Explorer 2.0 (ZEULAB S.L., Zaragoza, Spain)	*G. stearothermophilus* *	Meat (pork, chicken, ovine, bovine, etc.), liver, kidney, eggs, feed, and blood	More than 50 antibiotics of 8 classes of antibiotics (β-lactams, tetracyclines, sulfonamides, macrolides, aminoglycosides, lincosamides, anasamycins, and sulfones) at or below EU MRLs	[124,134]
Charm KIS (Charm Sciences, Lawrence, MA, USA)	*G. stearothermophilus* *	Fresh or frozen/thawed kidney tissue and muscle tissue (bovine, porcine, caprine, poultry, and ovine). Adaptable to water, feed extracts, poultry serum, and live animal urine	5 classes of antibiotics at or near kidney US or EU MRLs	[122,135]
No	*G. stearothermophilus* ATCC 12980	Muscle (porcine, bovine, poultry, and fish)	β-lactams, tetracyclines, macrolides, and sulfonamides in fish, porcine, bovine, and poultry muscle, and minoglycosides and lincosamides in fish muscle at or below EU MRLs	[17]
No	*G. stearothermophilus* C953	Meat	β-lactams, tetracyclines, aminoglycosides, and macrolides at or below EU MRLs	[18]
No	*G. stearothermophilus* C953	Milk, eggs, and honey with previous heating treatment	β-lactams, aminoglycosides, macrolides, and lincosamides in milk and eggs at or below EU MRLs. Tetracyclines, quinolones, and sulfonamides in milk above EU MRLs	[99]
No	*G. stearothermophilus* ATCC 12980	Milk	β-lactams, aminoglycosides (gentamicin, neomycin), macrolides (tylosin, tilmicosin), and sulfonamides at or below EU MRLs. Tetracyclines, streptomycin, dihydrostreptomycin, kanamycin, spectinomycin, erythromycin, spiramycin, sulfadimidine, and lincomycin above EU MRLs.	[118]
No	*G. stearothermophilus* ATCC 7953	Milk	β-lactams, tetracyclines, sulfonamides, and lincosamides (penicillin G, lincomycin, tylosin, neomyxin, and gentamicin) at or below Chinese MRLs. Kanamycin, streptomycin, and enrofloxacin at concentrations higher than Chinese MRLs	[100]

An asterisk indicates that the identity of the strain is confidential.

**Table 2:** Ethanol production at specified end-point time and productivity by different *(Para)Geobacillus* strains, either alone or in co-culture with other microorganisms, from various agri-food residues of differing compositions using a CBP approach.

Agri-Food Residue	% Carbohydrates and Type	Ethanol Production (g/L)	Productivity (g/L/h)	Microorganisms	References
Palm kernel cake hydrolysate (5%)	42–57% (hexoses: mannose and glucose; trace amounts of pentoses)	9.9 (48 h)	0.21	*P. thermoglucosidasius* TM242	[151]
Wheat straw (1%)	67–90% (cellulose, hemicellulose, lignin)	1.8 (24 h)	0.08	*P. thermoglucosidasius* LS242	[150]
		3.9 (24 h)	0.16	*P. thermoglucosidasius* BZ243	
		3.4 (24 h)	0.14	*P. thermoglucosidasius* BZ244	
Cafeteria food waste (20%)	56% (mainly starch sugars and cellulose and hemicellulose)	9.7 (48 h)	0.20	*P. thermoglucosidasius* ATCC 43742	[166]
		18.4 (120 h)	0.15	*P. thermoglucosidasius* ATCC 43742 and *T. ethanolicus* ATCC 31938	
Corn stover (1%)	79.9% (mainly cellulose and hemicellulose)	3.7 (72 h)	0.05	*Geobacillus* sp. DUSELR13 and *P. thermoglucosidasius* ATCC 43742	[152]
Bean curd refuse (1%)	35% (cellulose, hemicellulose, and pectin)	1.2 (48 h)	0.02	*Geobacillus* kpuB3 and *Thermoanaerobacterium* kpu04	[163]

**Table 3:** Commercially available enzymes, or those with proven potential applications in the food industry, produced by (*Para*)*Geobacillus* spp.

Enzyme	Strains	Food Industry Applications	References
α-amylase	*G. thermodenitrificans*	Reduction in staling, extension of shelf life, and/or retardation of retrogradation in cereal-based dough, rice, or pasta processing	[21]
	*G. stearothermophilus*	Starch processing, baking, brewing, and production of other cereal-based beverages	[214]
	*G. stearothermophilus*	Retardation of starch retrogradation, preservation of crumb softness, and preservation of elasticity	[215]
	*G. stearothermophilus*	Starch saccharification and improvement of bread shelf life and quality	[216]
Amylopullulanase	*G. thermoleovorans* NP33	Starch saccharification and improvement of bread shelf life, texture, and volume	[217]
Xylanase	*P. galactosidasius* BS61, *G. vulcani* GS90, and *Geobacillus* sp. TF16	Clarification of juices	[218,219,220]
	*Geobacillus* sp. TF16	Increase in rise rate of dough	[218]
β-Galactosidase	*G. kaustophilus* ATCC 8005	Production of low-lactose and lactose-free milk products	[26]
Metalloendopeptidase	*G. stearothermophilus*	Synthesis of phenylalanine (precursor of aspartame)	[31]
Protease	*G. stearothermophilus* CAU209	Production of antihypertensive whey protein hydrolysates	[221]

## Data Availability

No new data were created or analyzed in this study. Data sharing is not applicable to this article.
